# Effective Connectivity within the Mesocorticolimbic System during Resting-State in Cocaine Users

**DOI:** 10.3389/fnhum.2016.00563

**Published:** 2016-11-09

**Authors:** Suchismita Ray, Xin Di, Bharat B. Biswal

**Affiliations:** ^1^Center of Alcohol Studies, Rutgers, The State University of New Jersey, PiscatawayNJ, USA; ^2^New Jersey Institute of TechnologyNewark, NJ, USA

**Keywords:** connectivity, cocaine, fMRI, mesocorticolimbic system, resting state connectivity

## Abstract

**Objective:** Although effective connectivity between brain regions has been examined in cocaine users during tasks, no effective connectivity study has been conducted on cocaine users during resting-state. In the present functional magnetic resonance imaging study, we examined effective connectivity in resting-brain, between the brain regions within the mesocorticolimbic dopamine system, implicated in reward and motivated behavior, while the chronic cocaine users and controls took part in a resting-state scan by using a spectral Dynamic causal modeling (spDCM) approach.

**Method:** As part of a study testing cocaine cue reactivity in cocaine users (Ray et al., 2015b), 20 non-treatment seeking cocaine-smoking (abstinent for at least 3 days) and 17 control participants completed a resting state scan and an anatomical scan. A mean voxel-based time series data extracted from four key brain areas (ventral tegmental area, VTA; nucleus accumbens, NAc; hippocampus, medial frontal cortex) within the mesocorticolimbic dopamine system during resting-state from the cocaine and control participants were used as input to the spDCM program to generate spDCM analysis outputs.

**Results:** Compared to the control group, the cocaine group had higher effective connectivity from the VTA to NAc, hippocampus and medial frontal cortex. In contrast, the control group showed a higher effective connectivity from the medial frontal cortex to VTA, from the NAc to medial frontal cortex, and on the hippocampus self-loop.

**Conclusions:** The present study is the first to show that during resting-state in abstaining cocaine users compared to controls, the VTA initiates an enhanced effective connectivity to NAc, hippocampus and medial frontal cortex areas within the mesocorticolimbic dopamine system, the brain’s reward system. Future studies of effective connectivity analysis during resting-state may eventually be used to monitor treatment outcome.

## Introduction

The mesocorticolimbic system has been associated with reward, motivation, and goal-directed behavior. Drugs of abuse enhance extracellular dopamine concentration in components of the mesocorticolimbic system, including the ventral striatum (nucleus accumbens, NAc), extended amygdala, hippocampus, anterior cingulate, prefrontal cortex, and insula, which are triggered by dopaminergic projections essentially from the ventral tegmental area (VTA; Jasinska et al., 2014). Based on earlier studies (Jay, 2003; Kelley, 2004; Nestler, 2005), although the mesocorticolimbic system responds to natural rewards such as food, water, and sex, drugs of abuse induce a larger response in this system than physiological stimuli. Past research suggests that the drugs of abuse “hijack” the neurobiological mechanisms by which the brain reacts to reward, creates reward-related memories, and summarizes action repertoires leading to the reward (Everitt and Robbins, 2005; Kalivas and O’Brien, 2008). According to Volkow et al. (2006, 2008), through repeated drug use, drug related cues become conditioned stimuli and evoke dopamine release and craving; and over time, the incentive salience of these cues is heightened (Robinson and Berridge, 1993). This phenomenon of heightened salience of the drug cues has been demonstrated in human neuroimaging studies by increased blood oxygenation level dependent (BOLD) activation in areas including the prefrontal cortex [medial prefrontal cortex (mPFC), orbital frontal cortex, dorsolateral prefrontal cortex], VTA, anterior cingulate cortex, insula, NAc, amygdala, and hippocampus in response to drug cues relative to neutral cues in chronic drug users (see Jasinska et al., 2014 for review).

A major focus of the recent neuroimaging studies has been to understand not just which individual brain locations are activated by drug cues, but how individual brain regions are integrated, i.e., functional connectivity. Functional connectivity has been examined in cocaine users in resting-state (Gu et al., 2010; Wilcox et al., 2011; Cisler et al., 2013; Ray et al., 2015a) and also when they performed tasks (a finger-tapping and an attention task; Tomasi et al., 2010; Hanlon et al., 2011). According to Fox and Raichle (2007), resting state functional connectivity, typically assessed by the correlation of spontaneous fluctuations of BOLD signals in different regions of the ‘resting’ brain, is believed to provide a measure of the brain’s functional organization. Resting state functional connectivity between the regions within the mesocorticolimbic system in cocaine users has been examined by Gu et al. (2010). Results showed that cocaine users compared to controls had a reduced functional connectivity within this system. However, functional connectivity studies are limited in that although they provide information about the interaction of brain regions of interest (ROIs), these studies do not assess how one region influences another.

Effective connectivity on the other hand refers to the causal influence that one brain region employs over another, and thus add an important information on the consequences of chronic drug use on the mesocorticolimbic system. To the best of our knowledge, only three functional magnetic resonance imaging (fMRI) effective connectivity studies have been done with cocaine users. As part of the study described here, we have reported effective connectivity among brain regions within the drug cue processing network using IMaGES (Ramsey et al., 2010), a Bayesian search algorithm, while chronic cocaine users viewed cocaine-related picture cues (Ray et al., 2015b). During cocaine cue exposure, cocaine users demonstrated a unique feed-forward effective connectivity pattern between the ROIs of the drug-cue processing network (amygdala→hippocampus→dorsal striatum→insula→medial frontal cortex, dorsolateral prefrontal cortex, anterior cingulate cortex) that was absent when the controls viewed the cocaine cues. Using a stochastic dynamic causal modeling (DCM) approach, Ma et al. (2014) showed that cocaine subjects differed from controls in that effective connectivity from inferior frontal cortex to striatum was less affected by an immediate working memory task in the cocaine compared to the control group, and the effective connectivity from middle frontal gyrus to the striatum was less affected by the delayed working memory task in the cocaine compared to the control group. And Ma et al. (2015) utilized an fMRI-based stochastic DCM to study the effective neuronal connectivity associated with response inhibition in cocaine dependent subjects, elicited under performance of a Go/NoGo task with two levels of NoGo difficulty (Easy and Hard). The DCM analysis revealed that prefrontal-striatal connectivity was influenced during the NoGo conditions for both groups. In cocaine dependent subjects, the effective connectivity from left anterior cingulate cortex to left caudate was more negative during the Hard NoGo conditions.

**FIGURE 1:**
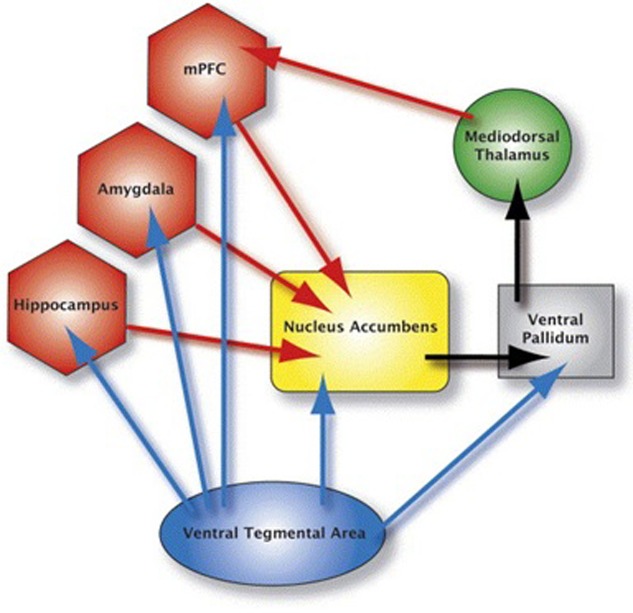
**This figure depicts the mesocorticolimbic system.** The blue arrows represent dopaminergic pathways: the red arrows represent glutamatergic pathways; black arrows represent GABAergic pathways. Brain areas anterior cingulate, insula, orbital frontal cortex and dorsolateral prefrontal cortex are not shown in the figure.

The goal of this study was to expand Gu et al.’ (2010) study by examining effective connectivity among regions within the mesocorticolimbic dopamine system (**Figure 1**) in cocaine users during resting-state, when there are no demands being placed, such as cognitive tasks or viewing drug cues. This provided a measure of baseline effective connectivity, utilizing baseline BOLD signal, within the mesocorticolimbic system in cocaine users (Liu et al., 2011). Since there is no demand on task, resting-state data unburden subject compliance, and training demands, and thus makes it interesting for studies of development and clinical populations. An analysis of baseline connectivity might shed light on the interpretation of prior research that has found an increased connectivity in cocaine users (vs. controls) in response to, for example, a cognitive task. Conceivably, such a finding might be due to a characteristically higher resting state level of connectivity for cocaine users; if true, then a conclusion that higher connectivity is due to a cognitive task would be called into question. In the present fMRI study, we examined effective connectivity in resting-brain, more specifically, between the brain regions within the mesocorticolimbic dopamine system while the chronic cocaine users took part in a resting-state scan. We collected resting-state fMRI data from cocaine smokers who were non-treatment seekers and were abstinent from cocaine use for 72 h and age-matched healthy controls with no experience with cocaine.

Although originally developed for task based fMRI (Friston et al., 2003), several methodological developments have made it possible to use DCM to model effective connectivity during resting-state (Daunizeau et al., 2012; Di and Biswal, 2014; Friston et al., 2014). One of the recent developments is to inverse DCM models at the frequency spectrum domain (Friston et al., 2014). In the current study, we applied this spectral DCM (spDCM) approach on resting-state fMRI data collected from chronic cocaine users and controls to examine effective connectivity among four key regions within the mesocorticolimbic dopamine system: VTA, NAc, hippocampus, and medial frontal cortex. We first set fully connected models for the two groups. We then adopted a Bayesian model reduction approach to identify optimal models for the two groups (Friston and Penny, 2011). More specifically, based on research conducted by Gu et al. (2010), we hypothesized that the cocaine group compared to the control group would show a decreased effective connectivity pattern between the four regions within the mesocorticolimbic dopamine system as a result of chronic cocaine use during resting-state.

## Materials and Methods

### Participants

Twenty (15M; 5F) non-treatment seeking chronic cocaine smokers abstaining from cocaine use for 72 h, and 17 (13M; 4F) age-, education-, and ethnic-background matched healthy control participants took part in the study (**Table 1**). The two groups did not significantly differ with regard to their age, education, alcohol use quantity, nicotine use frequency and quantity, and caffeine use frequency and quantity.

**Table 1:** Demographic and substance use information for cocaine users and controls.

	Cocaine (*n*=20)	Control (*n*=17)		
	Mean, Range (*SD*)	Mean, Range (*SD*)	*t*-stats	*p*
Age (years)	46 (6.4)	46 (7)	0.10	0.92
Education (years)	13.4 (2.4)	13.5 (2.1)	-0.17	0.86
**Race/Ethnicity**				
Caucasian	7	5		
African American	11	11		
Hispanic	2	1		
Female (*n*)	5	4		
**Cocaine Use by All Users**				
Frequency (days/week)	3, 2–6 (1.2)	NA		
Duration of use (years)	16, 3–34 (8)	NA		
Money spent ($/week)	$220, $70–550 (131)	NA		
**Cocaine Use by Non-cocaine dependent/abusers**				
Frequency (days/week)	3, 2–6 (1.5)			
Duration of use (years)	9, 3–19 (6)			
Money spent ($/week)	$172, $80–350 (93)			
**Alcohol Use**				
Frequency (days/month)	1.9, 1–2.5 (0.55)	4.0, 2.5–6.5 (1.4)	-4.89	0.00^∗^
Quantity (drinks/occasion)	2.1, 1–3.5 (0.92)	1.7, 1–2 (0.42)	0.92	0.37
Drinkers (#)	13	6		
**Nicotine Use**				
Frequency (days/week)	5.1, 1–7 (2.3)	5.7, 3–7 (2.3)	-0.40	0.70
Quantity (cigarettes/day)	6.3, 1.5–13 (3.0)	2.8, 2.5–3 (0.29)	2.00	0.07
Smokers (#)	13	6		
**Caffeine Use**				
Frequency (days/week)	4.4, 1–7 (2.5)	3.6, 1–7 (2.4)	0.78	0.44
Quantity (cups/day)	1.3, 1–2 (0.43)	1.3, 1–4 (0.90)	0.26	0.80
Caffeine users (#)	13	11		
**Clinical Characteristics**				
DSM-IV-R cocaine dependence	10	NA		
DSM-IV-R cocaine abuse	3	NA		
Cocaine non-dependent/abusers	7			

*^∗^Denotes significant group difference.*

The main inclusion criteria for the study participants included English as their first language, no report of childhood learning disability or special education, right handedness, and near 20/20 vision (or corrected). The main exclusion criteria for the study participants included serious medical conditions, a history of psychiatric or neurological disorder or treatment, lifetime diagnosis of any substance use disorder of the prospective participant’s biological mother (to rule out prenatal exposure effects), MRI contraindications, alcohol abuse and dependence including past dependence on alcohol, and for women, pregnancy. Participants were excluded if they reported any history of anxiety or depression in their recent past.

Participants were included in the cocaine group if they currently spent a minimum of $70 per week on cocaine and had a history of smoking cocaine for at least two times per week for the past 6 months (assessed by self-report). Participants in the cocaine group were instructed to abstain from cocaine for at least 72 h before their study appointment. The primary current drug of choice for the cocaine group was cocaine and they did not meet a DSM-IV-TR diagnosis of abuse or dependence for any other drugs, as confirmed by SCID (First et al., 1997). Half of the cocaine users did not meet DSM-IV-TR criteria for cocaine dependence, and seven did meet criteria for abuse or dependence. Ten out of 20 cocaine users never tried any other drugs in their lifetime and nine others experimented with marijuana once or two times in their lifetime ranging from 15 to 30 years back. Only one used marijuana one time in his/her lifetime 3 weeks before the study. Participants were included in the control group if they did not have any current or past drug use history and had no alcohol abuse history in their first degree family members. Ten out of 17 controls never tried any drugs in their lifetime and seven others experimented with marijuana once or two times in their lifetime ranging from 30 to 43 years back. Family history of alcohol abuse was assessed by using a semi-structured diagnostic instrument called Family History Assessment Module (Cloninger and Reich, 1991). None of the participants in the cocaine or in the control group reported any history of anxiety or depression during the past 2 weeks on the day of the telephone screening interview which took place within 7 days of the study.

On the day of the study, all participants gave written informed consent and took a urine screen to rule out pregnancy in women, and to ensure negative urine toxicology for cocaine, methamphetamine, THC, opiate and benzodiazepines (One Step Multi-Drug Screen Test Panel). Abstinence from alcohol was confirmed with a breathalyzer. At the end of the study, participants were compensated with a gift certificate worth $100 for their participation and were paid for their transportation expenses (Ray et al., 2015b). This research was approved by the Rutgers University Institutional Review Board.

### Procedure

Each participant completed a resting-state scan and a high resolution anatomical MPRAGE (magnetization-prepared rapid acquisition with gradient echo) scan. During resting-state scan, participants were instructed to lie quietly without any movements while they visually fixated on a cross for 6 min. All participants completed resting-state scan first and then took part in the cue exposure task. All participants were administered a cocaine-craving questionnaire (CCQ-Brief; Sussner et al., 2006) before the resting-state scan started. They had to rate their craving for cocaine on a seven-point scale (1 = Strongly Disagree, 7 = Strongly Agree).

### Image Acquisition

Imaging data were collected using a 3T Siemens Trio head-only fMRI scanner equipped with a standard Siemens head coil. While participants visually fixated on the cross, T2^∗^-weighted echo planar images were acquired (35 axial slices, voxel size 3 mm × 3 mm × 3 mm, interslice gap 1 mm, matrix size 64 mm × 64 mm, *FOV* = 192 mm, *TR* = 2000 ms, *TE* = 25 ms, flip angle = 90°) covering the entire brain. A sagittal T1-weighed structural scan (*TR* = 1900 ms, *TE* = 2.52 ms, matrix = 256 × 256, *FOV* = 256 mm, voxel size 1 mm × 1 mm × 1 mm, 176 1-mm slices with 0.5 mm gap) was acquired in order to co-register it with the fMRI data (Ray et al., 2015b).

### ROIs Selection

**FIGURE 2:**
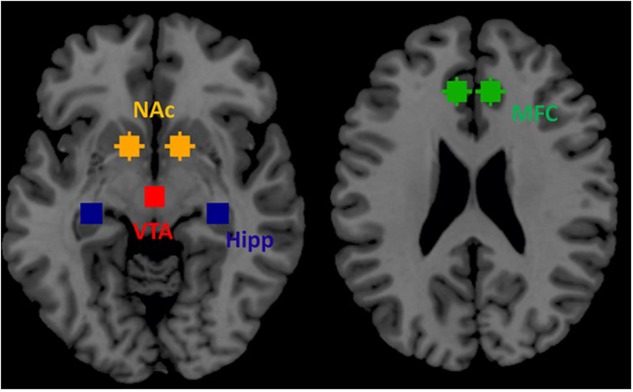
**Four regions of interest (ROIs) within the mesocorticolimbic system that were used for spDCM analysis**.

Based on prior publications in the field of addiction (see section 3.3.1. of Jasinska et al., 2014) we selected four ROIs within the mesocorticolimbic dopamine system as key nodes for effective connectivity analysis during resting-state. These four ROIs included VTA, NAc, hippocampus and medial frontal cortex. We selected these four regions: (1) VTA because regions within the mesocorticolimbic system are innervated by dopaminergic projections predominantly from the VTA, (2) VTA directly sends its projection to NAc (ventral striatum) implicated in reward and motivation, (3) hippocampus is responsible for memory related to past drug use, and (4) medial frontal cortex is implicated in continuation of drug seeking behavior (Jasinska et al., 2014). These four regions well represent mesocorticolimbic system (**Figure 2**).

### Data Preprocessing

For each participant, in the first step, first five time-points were removed from that participant’s BOLD fMRI data to account for T1-relaxation effects. In the next step, the participant’s BOLD fMRI data were motion corrected with respect to the mean image of that participant. Following motion correction, each participant’s BOLD fMRI data were co-registered to the anatomical images for that participant. Following co-registration, each participant’s anatomical images were segmented into gray matter, white matter, cerebrospinal fluid (CSF) images and the deformation fields were derived to transform each participant’s BOLD fMRI data into the MNI standard space. Lastly, 24 head motion parameters (Friston et al., 1996), the first five principle components of signals from white matter, and first five principle components of signals from CSF were regressed out for every voxel using linear regression.

In order to study effective connectivity patterns of the mesocorticolimbic dopamine system, we defined a total of four brain regions based on Jasinska et al. (2014). For each cocaine participant, a mean voxel based time series was extracted from each of these four ROIs (bilateral) using the AFNI program ‘3dmaskave’, and used as input to the spDCM analysis in modeling the causal interactions between the ROIs during resting-state. For these four ROIs, the mean voxel based time series for the right brain area (i.e., right hippocampus) and the left brain area (i.e., left hippocampus) were averaged to create the mean voxel based time series for that brain area (hippocampus). A mean voxel-based time series data extracted from the same ROIs during resting-state from the control participants were used as input to the spDCM program to generate an additional spDCM analysis output.

### Dynamic Causal Modeling

SPM 12 (with updates 6685) was used to perform spDCM analysis. For each subject, we first built a DCM with all endogenous connectivity specified (full model). All other types of connectivity, i.e., B, C, and D parameters, were set as zero. We used spectrum DCM framework to inverse the model for each subject (Friston et al., 2014). We next employed a network discovery procedure to optimize the DCMs for each group, separately (Friston and Penny, 2011). This procedure tests all the models nested in the full model, and chose the model with highest posterior probability. We then adopted Bayesian parameter averaging (BPA) approach to obtain model parameters for each group, separately (Razi et al., 2015). To compare connectivity parameters between the two groups, we compared model parameters from the full models between the two groups by using the BPA approach. Group differences in connectivity were identified using false discovery rate (FDR) at *p* < 0.05 correcting for the total 16 (4 × 4) connectivity parameters.

## Results

### Motion Comparison

All participants met the motion threshold (0.5 mm) as set for the study. That is, for all participants, the mean frame-wise displacement was less than 5 mm. A group level unpaired *t*-test revealed that groups did not differ in mean frame-wise displacement (*p* = 0.8120). The average mean frame-wise displacement was 0.177 mm in the cocaine group and 0.185 mm in the control group.

### Craving Results

For each participant, craving scores were obtained (Sussner et al., 2006) before the resting state scan. Results showed that cocaine users did not show significantly higher craving rating compared to controls [*t*(35) = 1.02, *p* = 0.31; 1.23 (*SD* = 1.03) vs. 1 (*SD* = 0)].

### Dynamic Causal Modeling

**FIGURE 3:**
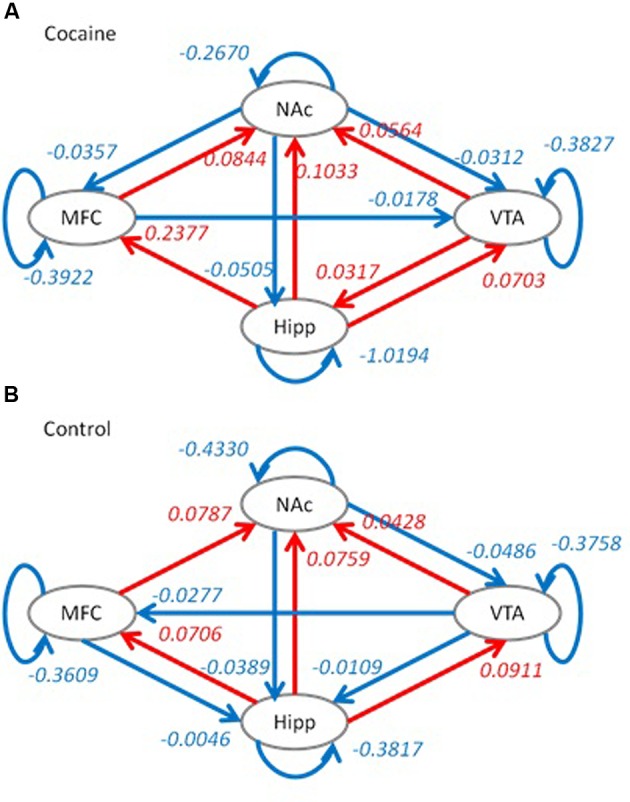
**Optimized dynamic causal models (DCMs) for the cocaine group (A)** and the control group **(B)**. Red color indicates positive effective connectivity, while blue color indicates negative effective connectivity. Numbers represent averaged effective connectivity strengths using Bayesian parameter averaging. Hipp, hippocampus; MFC, medial frontal cortex; VTA, ventral tegmental area; NAc, nucleus accumbens.

Model optimization procedure gave slightly different model structures for the two groups. For the cocaine group, the effective connectivity from the VTA to medial frontal cortex and effective connectivity from the medial frontal cortex to hippocampus were removed. While for the control group, the effective connectivity from the NAc to medial frontal cortex and effective connectivity from medial frontal cortex to VTA were removed. The effective connectivity structures along with averaged connectivity parameters for the two groups are shown in **Figure 3**.

**FIGURE 4:**
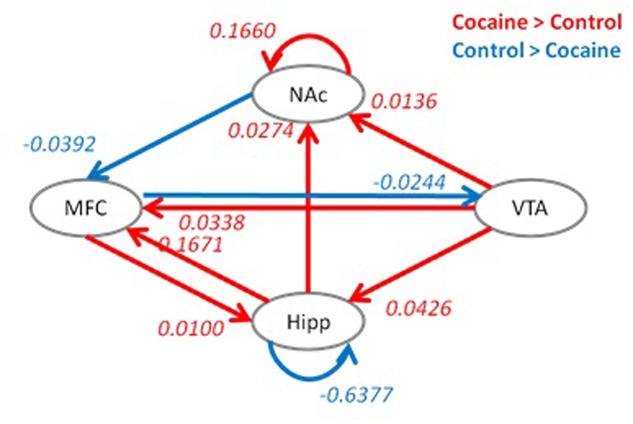
**Group differences in effective connectivity parameters between the cocaine and control groups.** Red indicates a greater effective connectivity in the cocaine group compared to the control group, and blue indicates a reduced effective connectivity in the cocaine group compared to the control group. Significant group differences were identified after false discovery rate (FDR) correction at *p* < 0.05. Hipp, hippocampus; MFC, medial frontal cortex; VTA, ventral tegmental area; NAc, nucleus accumbens.

Group differences in effective connectivity parameters of the full model between the two groups are shown in **Figure 4**. Compared to the control group, the cocaine group had higher effective connectivity for seven connections (red arrows), and reduced effective connectivity for three connections (blue arrows). The cocaine group showed higher effective connectivity from the VTA to NAc, hippocampus and medial frontal cortex. In addition, the effective connectivity from the hippocampus to NAc, the reciprocal effective connectivity between the hippocampus and medial frontal cortex, and the self-effective connectivity of the NAc also showed a greater effective connectivity in the cocaine group compared to the control group. In contrast, the control group showed a higher effective connectivity from the medial frontal cortex to VTA, from the NAc to medial frontal cortex, and on the hippocampus self-loop.

## Discussion

The objective of this fMRI study was to compare effective connectivity among four key brain regions within the mesocorticolimbic dopamine system in chronic cocaine users to healthy controls during resting-state, when there are no demands being placed, such as cognitive tasks or viewing drug cues. This provided us a measure of baseline effective connectivity within the mesocorticolimbic dopamine system in chronic users of cocaine which is not available by measuring effective connectivity while the cocaine users perform a task. To examine effective connectivity, we employed one of the recently developed DCM models which utilizes the frequency spectrum domain (spDCM; Friston et al., 2014).

According to Jasinska et al. (2014), drugs of abuse enhance extracellular dopamine concentration in components of the mesocorticolimbic system, including the NAc, extended amygdala, hippocampus, anterior cingulate, prefrontal cortex, and insula, which are triggered by dopaminergic projections essentially from the VTA (Jasinska et al., 2014). Since VTA sends projections to multiple areas within the mesocorticolimbic system, we decided that these connections would provide a good way to compare cocaine users with controls during resting-state. Our results provided a mixed support of our hypothesis. More specifically, group differences in effective connectivity pattern revealed that the control group compared to the cocaine group showed a higher effective connectivity from the medial frontal cortex to VTA, from the NAc to medial frontal cortex, and on the hippocampus self-loop, consistent with our hypothesis. However, contrary to our hypothesis, the cocaine group compared to the control group showed a greater effective connectivity from the VTA to all three other areas within the mesocorticolimbic dopamine system, that is, NAc, hippocampus and medial frontal cortex (**Figure 4**). Perhaps neuroplasticity within the mesocorticolimbic dopamine reward system as a result of chronic cocaine use may account for these differences in effective connectivity patterns between cocaine users and controls. We speculate that the higher effective connectivity from the medial frontal cortex to VTA and from the NAc to medial frontal cortex represent better cortical and subcortical communications in controls compared to cocaine users. More specifically, higher effective connectivity from the medial frontal cortex to VTA demonstrates control participants’ higher cortical cognitive control on subcortical region (VTA; Ridderinkhof et al., 2004) that may have implications for reducing drug seeking behavior.

We further speculate that may be the effective connectivity alterations throughout the mesocorticolimbic reward system revealed during resting-state in chronic users of cocaine play a role in maintaining problematic drug use. An enhanced causal influence of VTA on NAc, hippocampus and medial frontal cortex in cocaine users compared to control is consistent with Jasinska et al. (2014), who suggested that drugs of abuse increase dopaminergic projections predominantly from the VTA to other areas within the mesocorticolimbic system.

The present study extends upon the previous research including research by Gu et al. (2010) by establishing for the first time that during resting-state in abstaining cocaine users, the VTA created an enhanced effective connectivity to NAc, hippocampus and medial frontal cortex in cocaine users compared to controls within the brain’s reward system. The present findings are, however, contrary to Gu et al. (2010) who showed a reduced functional connectivity between regions within the mesocorticolimbic system, including between VTA and ventral striatum, between amygdala and mPFC, and between hippocampus and dorsal mPFC during resting-state in cocaine users compared to controls. Yet the majority of participants in Gu et al.’ (2010) study did not abstain from cocaine during the resting-state scan, so their findings may reflect, in part, the acute effects of cocaine, which change resting-state functional connectivity.

Resting-state functional connectivity has been linked to self-monitoring and introspective processes (Eryilmaz et al., 2011). We speculate that, during the resting-state scan, a greater effective connectivity from the VTA to hippocampus within the mesocorticolimbic dopamine system in cocaine users compared to controls may reflect persistent thoughts of the cocaine users’ long-term memory of drug use (Tiffany, 1990; Spaniol et al., 2009; Jasinska et al., 2014), consistent with the activation of hippocampus by VTA. We also speculate that an enhanced effective connectivity from the VTA to medial frontal cortex in cocaine users compared to controls may reflect activation of decision making and motivated behavior related to continued drug use (Balleine et al., 2007; Jasinska et al., 2014). In future studies, participants might be interviewed post-scan to understand the content of their thoughts while they were inside the scanner. As potential system-level biomarkers of chronic cocaine use, the alterations within the mesocorticolimbic dopamine system may be usefully applied in treatment development and monitoring treatment outcome. It would be particularly useful to examine whether therapeutic interventions change the enhanced effective connectivities that were found in cocaine users within this system which may imply a positive treatment outcome.

Next, we would like to mention a couple of limitations of this study. First, although we matched the cocaine smoking and control groups based on their age, educational and ethnic/racial background, controls drank significantly more alcohol than the cocaine-using group (**Table 1**). However, importantly, alcohol use was still very low for both groups (<1 drink/day), therefore, was unlikely to affect our findings. This does, however, restrict our conclusions to a ‘pure’ cocaine-using group and may not be generalizable to cocaine users who abuse alcohol as well. Second, we acknowledge that we had a small sample size. There were only five female cocaine smokers in our study, thus, we could not investigate any potential sex differences in our resting-state study outcome. Third, due to limitation of the BPA approach that does not allow us to put alcohol and nicotine usage as covariates in group level analysis, we could not use alcohol use frequency and nicotine use quantity as covariates. However, alcohol usage frequency was actually significantly lower in the cocaine group than the control group, and conversely nicotine use quantity was higher in the cocaine group than the control group (non-significant). Despite these limitations, the results of the present study provide a model of effective connectivity among four regions within the mesocorticolimbic dopamine system during resting-state in individuals who are chronic users of cocaine. An important issue in interpreting results of a cross-sectional study, such as ours, is whether differences between groups are a consequence of chronic drug use or alternatively, reflect pre-existing differences that predispose some individuals to addiction. This can be investigated in future studies that will utilize a longitudinal design.

To conclude, the present study is the first to show that during resting-state in abstaining cocaine users compared to controls, the VTA initiates an enhanced effective connectivity to NAc, hippocampus and medial frontal cortex areas within the mesocorticolimbic dopamine system, the brain’s reward system. Future studies of effective connectivity analysis during resting-state may eventually be used to monitor treatment outcome.

## Author Contributions

SR, designed and ran the study, and wrote the manuscript. XD, conducted the analysis, and wrote part of the results section. BB helped with the design, addressed reviewers’ concerns, and edited the manuscript.

## Conflict of Interest Statement

The authors declare that the research was conducted in the absence of any commercial or financial relationships that could be construed as a potential conflict of interest.

## References

[B1] Balleine B. W. Delgado M. R. Hikosaka O. (2007). The role of the dorsal striatum in reward and decision-making. *J. Neurosci.* 27 8161–8165. 10.1523/JNEUROSCI.1554-07.2007 17670959 PMC6673072

[B2] Cisler J. M. Elton A. Kennedy A. P. Young J. Smitherman S. Andrew James G. (2013). Altered functional connectivity of the insular cortex across prefrontal networks in cocaine addiction. *Psychiatry Res.* 213 39–45. 10.1016/j.pscychresns.2013.02.007 23684980 PMC3708551

[B3] Cloninger R. Reich T. (1991). *Family History Assessment Module. Based on HELPER Family Data Interview.* St. Louis, MO: Washington University School of Medicine.

[B4] Daunizeau J. Stephan K. E. Friston K. J. (2012). Stochastic dynamic causal modelling of fMRI data: should we care about neural noise? *Neuroimage* 62:464481 10.1016/j.neuroimage.2012.04.061 PMC3778887 22579726

[B5] Di X. Biswal B. B. (2014). Identifying the default mode network structure using dynamic causal modeling on resting-state functional magnetic resonance imaging. *Neuroimage* 86 53–59. 10.1016/j.neuroimage.2013.07.071 23927904 PMC3947265

[B6] Eryilmaz H. Van De Ville D. Schwartz S. Vuilleumier P. (2011). Impact of transient emotions on functional connectivity during subsequent resting state: A wavelet correlation approach. *Neuroimage* 54 2481–2491. 10.1016/j.neuroimage.2010.10.021 20955802

[B7] Everitt B. J. Robbins T. W. (2005). Neural systems of reinforcement for drug addiction: from actions to habits to compulsion. *Nat. Neurosci.* 8 1481–1489. 10.1038/nn1579 16251991

[B8] First M. B. Spitzer R. L. Gibbon M. Williams J. B. W. (1997). *Structured Clinical Interview for DSM-IV Axis I Disorders-Patient Edition (SCID-I/P, Version 2.0* 4/97 revision). New York, NY: New York State Psychiatric Institute.

[B9] Fox M. D. Raichle M. E. (2007). Spontaneous fluctuations in brain activity observed with functional magnetic resonance imaging. *Nat. Rev. Neurosci.* 8 700–711. 10.1038/nrn2201 17704812

[B10] Friston K. Penny W. (2011). Post hoc Bayesian model selection. *Neuroimage* 56 2089–2099. 10.1016/j.neuroimage.2011.03.062 21459150 PMC3112494

[B11] Friston K. J. Harrison L. Penny W. (2003). Dynamic causal modelling. *Neuroimage* 19 1273–1302. 10.1016/S1053-8119(03)00202-7 12948688

[B12] Friston K. J. Kahan J. Biswal B. Razi A. (2014). A DCM for resting state fMRI. *Neuroimage* 94 396–407. 10.1016/j.neuroimage.2013.12.009 24345387 PMC4073651

[B13] Friston K. J. Williams S. Howard R. Frackowiak R. S. Turner R. (1996). Movement-related effects in fMRI time-series. *Magn. Reson. Med.* 35 346–355. 10.1002/mrm.1910350312 8699946

[B14] Gu H. Salmeron J. B. Ross J. T. Geng X. Zhan W. Stein E. A. (2010). Mesocorticolimbic circuits are impaired in chronic cocaine users as demonstrated by resting-state functional connectivity. *Neuroimage* 53 593–601. 10.1016/j.neuroimage.2010.06.066 20603217 PMC2930044

[B15] Hanlon C. A. Wesley M. J. Stapleton J. R. Laurienti P. J. Porrino L. J. (2011). The association between frontal-striatal connectivity and sensorimotor control in cocaine users. *Drug Alcohol Depend.* 115 240–243. 10.1016/j.drugalcdep.2010.11.008 21193273 PMC3499027

[B16] Jasinska A. J. Stein E. A. Kaiser J. Naumer M. J. Yalachkov Y. (2014). Factors modulating neural reactivity to drug cues in addiction: a survey of human neuroimaging studies. *Neurosci. Biobehav. Rev.* 38 1–16. 10.1016/j.neubiorev.2013.10.013 24211373 PMC3913480

[B17] Jay T. M. (2003). Dopamine: a potential substrate for synaptic plasticity and memory mechanisms. *Prog. Neurobiol.* 69 375–390. 10.1016/S0301-0082(03)00085-6 12880632

[B18] Kalivas P. W. O’Brien C. (2008). Drug addiction as a pathology of staged neuroplasticity. *Neuropsychopharmacology* 33 166–180. 10.1038/sj.npp.1301564 17805308

[B19] Kelley A. E. (2004). Memory and addiction: shared neural circuitry and molecular mechanisms. *Neuron* 44 161–179. 10.1016/j.neuron.2004.09.016 15450168

[B20] Liu X. Zhu X. Chen W. (2011). Baseline BOLD correlation predicts individuals’ stimulus-evoked BOLD responses. *Neuroimage* 54 2278–2286. 10.1016/j.neuroimage.2010.10.001 20934521 PMC3006639

[B21] Ma L. Steinberg J. L. Cunningham K. Lane S. Bjork J. Neelakantan H. (2015). Inhibitory behavioral control: a stochastic dynamic causal modeling study comparing cocaine dependent subjects and controls. *Neuroimage* 7 837–847. 10.1016/j.nicl.2015.03.015 26082893 PMC4459041

[B22] Ma L. Steinberg J. L. Hasan K. M. Narayana P. A. Kramer L. A. Moeller F. G. (2014). Stochastic dynamic causal modeling of working memory connections in cocaine dependence. *Hum. Brain Mapp.* 35 760–778. 10.1002/hbm.22212 23151990 PMC4440319

[B23] Nestler E. J. (2005). Is there a common molecular pathway for addiction? *Nat. Neurosci.* 8 1445–1449. 10.1038/nn1578 16251986

[B24] Ramsey J. D. Hanson S. J. Hanson C. Halchenko Y. O. Poldrack R. A. Glymour C. (2010). Six problems for causal inference from fMRI. *Neuroimage* 49 1545–1558. 10.1016/j.neuroimage.2009.08.065 19747552

[B25] Ray S. Gohel S. Biswal B. B. (2015a). Altered functional connectivity strength in abstinent chronic cocaine smokers compared to healthy controls. *Brain Connect.* 5 476–486. 10.1089/brain.2014.0240 26005203 PMC4601553

[B26] Ray S. Haney M. Hanson C. Biswal B. Hanson S. J. (2015b). Modeling causal relationship between brain regions within the drug-cue processing network in chronic cocaine smokers. *Neuropsychopharmacology* 40 2960–2968. 10.1038/npp.2015.150 26038158 PMC4864631

[B27] Razi A. Kahan J. Rees G. Friston K. J. (2015). Construct validation of a DCM for resting state fMRI. *Neuroimage* 106 1–14. 10.1016/j.neuroimage.2014.11.027 25463471 PMC4295921

[B28] Ridderinkhof K. R. Ullsperger M. Crone E. A. Nieuwenhuis S. (2004). The role of the medial frontal cortex in cognitive control. *Science* 306 443–447. 10.1126/science.1100301 15486290

[B29] Robinson T. E. Berridge K. C. (1993). The neural basis of drug craving: an incentive sensitization theory of addiction. *Brain Res. Rev.* 18 247–291. 10.1016/0165-0173(93)90013-P 8401595

[B30] Spaniol J. Davidson P. S. Kim A. S. Han H. Moscovitch M. Grady C. L. (2009). Event-related fMRI studies of episodic encoding and retrieval: meta-analyses using activation likelihood estimation. *Neuropsychologia* 47 1765–1779. 10.1016/j.neuropsychologia.2009.02.028 19428409

[B31] Sussner B. D. Smelson D. A. Rodrigues S. Kline A. Losonczy M. Ziedonis D. (2006). The validity and reliability of a brief measure of cocaine craving. *Drug Alcohol Depend.* 83 233–237. 10.1016/j.drugalcdep.2005.11.022 16384655

[B32] Tiffany S. T. (1990). A cognitive model of drug urges and drug-use behavior: role of automatic and nonautomatic processes. *Psychol. Rev.* 97 147–168. 10.1037/0033-295X.97.2.147 2186423

[B33] Tomasi D. Volkow N. D. Wang R. Carrillo H. J. Maloney T. Alia-Klien N. (2010). Disrupted functional connectivity with dopaminergic midbrain in cocaine abusers. *PLoS ONE* 5:e10815 10.1371/journal.pone.0010815 PMC2876035 20520835

[B34] Volkow N. D. Wang G. J. Telang F. Fowler J. S. Logan J. Childress A. R. (2006). Cocaine cues and dopamine in dorsal striatum: mechanism of craving in cocaine addiction. *J. Neurosci.* 26 6583–6588. 10.1523/JNEUROSCI.1544-06.2006 16775146 PMC6674019

[B35] Volkow N. D. Wang G. J. Telang F. Fowler J. S. Logan J. Childress A. R. (2008). Dopamine increases in striatum do not elicit craving in cocaine abusers unless they are coupled with cocaine cues. *Neuroimage* 39 1266–1273. 10.1016/j.neuroimage.2007.09.059 18024160 PMC3170090

[B36] Wilcox C. E. Teshiba T. M. Merideth F. Ling J. Mayer A. R. (2011). Enhanced cue reactivity and fronto-striatal functional connectivity in cocaine use disorders. *Drug Alcohol Depend.* 115 137–144. 10.1016/j.drugalcdep.2011.01.009 21466926 PMC3090708

